# Administration of Salicylates

**Published:** 1886-09-20

**Authors:** 


**Administration of the Salicylates.**—The *Therapeutic Gazette* (editorial) says that clinical experience seems to us to have proven that the salicylates are almost as much of a specific in rheumatism as is quinine in intermittent fever. The cases are further parallel in the facts that precisely as we have no knowledge of the nature of the malarial poison, and the way in which it affects the system, so have we no knowledge of the nature of the rheumatic poison and its relation to the system; if, indeed, rheumatism be due to the presence of the poison, and be not after all, a neurosis.

The best method of administering quinine in malarial fever is even yet one concerning which physicians are not agreed; but, in our opinion, the right method is not to give for a length of time 6, 8, or 10 grains a day of the cinchona alkaloid, but to employ it in overwhelming dose, so as to at once abort the intermittent paroxysm. We are much inclined to believe that the proper use of the salicylates is in the same way, and that many of the failures that occur with it in rheumatism are due to an incorrect method of administration. We have always obtained the best results by giving the remedy for thirty-six hours in doses as large as can be borne, then ceasing medication for one, two, or three days, and again giving a very large dose.

There is little or no danger from the use even of very large doses of the salicylate, provided that these doses be separated from one another so that no accumulation of the salt can occur in the system.

There is one contradiction to the use of salycilates which we have not seen much dwelt upon by writers; namely, chronic congestion or catarrh of the middle ear. The tinnitus aurium which salicylic acid produces has been proven to be the result of an intense irritative congestion of the middle and internal ear. A notable deafness is always present in persons who are fully under its action, and when the inner portions of the aural apparatus are diseased this deafness is greatly exaggerated. More important than the mere temporary inconvenience and distress which is produced in these cases is the liability of permanently intensifying the existing deafness. The circumstances must be very urgent which would warrant the exhibition of full doses of the salicylates to the victims of chronic aural lesions.—*Epitome.*

